# Supplementary figures and images for: bHLH transcription factors cooperate with chromatin remodelers to regulate cell fate decisions during Arabidopsis stomatal development
Source: PLoS Biol. 2024 Aug 16;22(8):e3002770. doi: 10.1371/journal.pbio.3002770 (PMC11357106; doi:10.1371/journal.pbio.3002770)

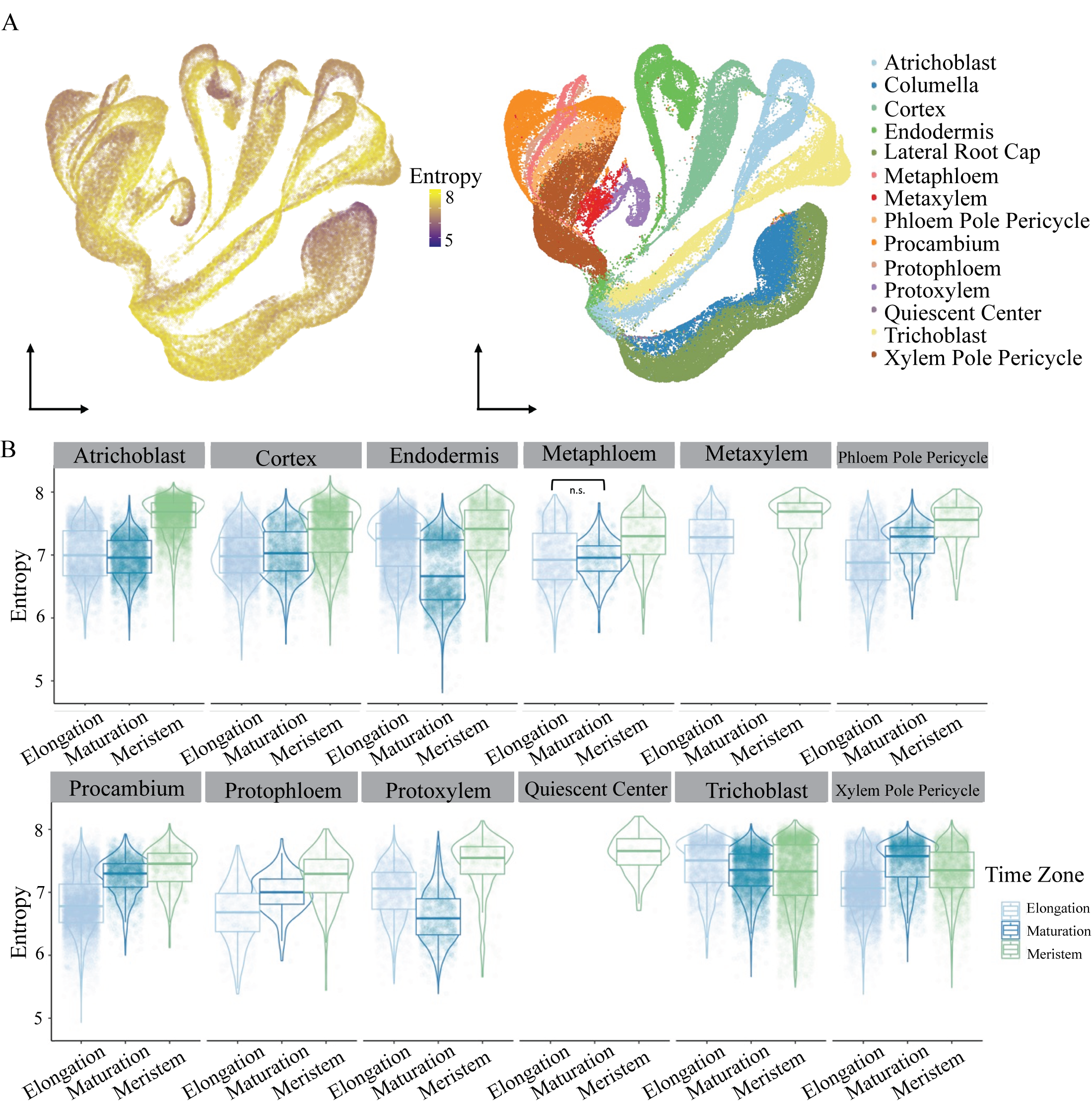

Supplement: S1 Fig — (A) Entropy scores (left) and cell identities (right) in root cells derived from scRNA-seq dataset in [26]. (B) Boxplot of entropy scores of different root cell types along a developmental gradient where meristem represents the least differentiated cells, followed by maturation and then elongation (most differentiated). The data underlying this figure can be found in S1 Data. (TIF) [file pbio.3002770.s001.tif]

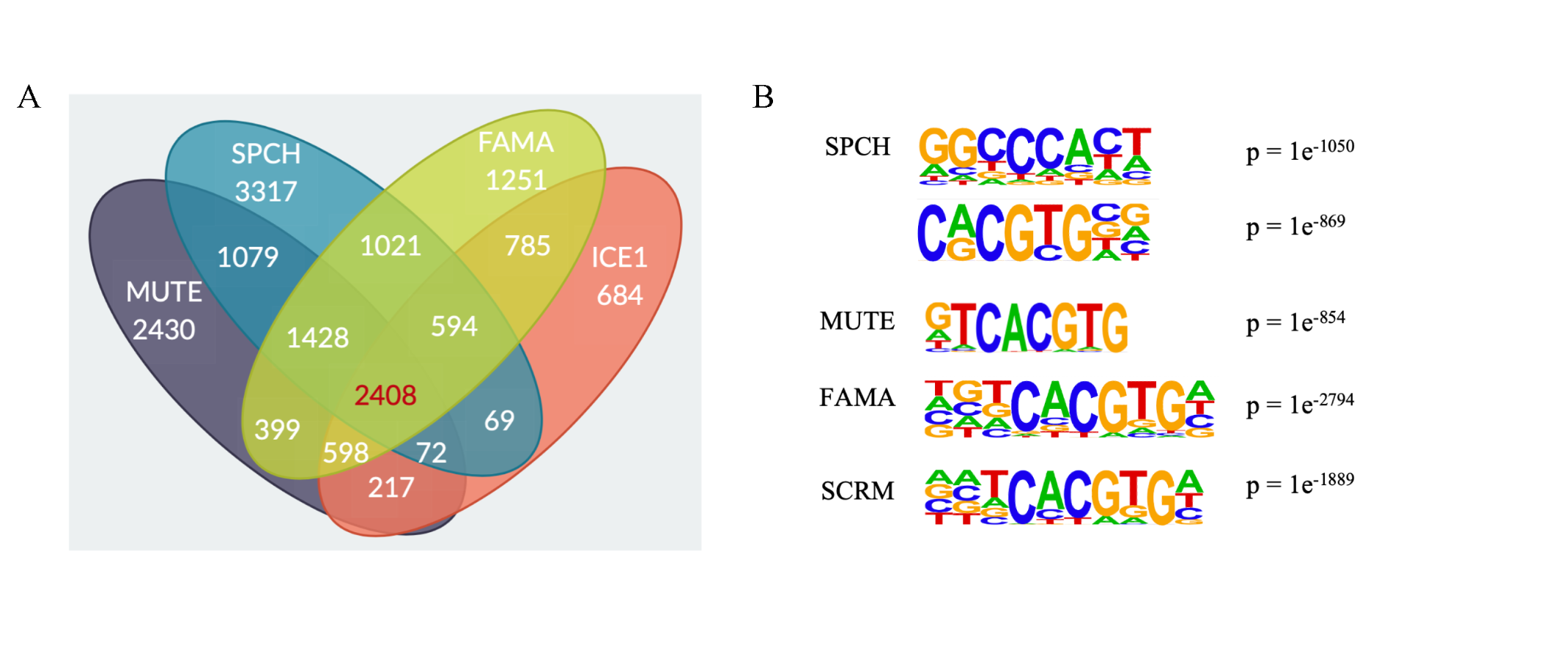

Supplement: S2 Fig — (A) A Venn diagram showing the overlap of ChIP-seq peaks among the stomatal bHLHs, data sources shown in S1 Data. (B) Top motifs in the stomatal bHLHs’ binding sites as determined by ChIP-seq of the respective bHLHs. Note that all factors recognize a G-box motif, but SPCH has an additional alternative preferred binding site. bHLH, basic helix–loop–helix; ChIP-seq, chromatin immunoprecipitation followed by deep sequencing; SPCH, SPEECHLESS. (TIF) [file pbio.3002770.s002.tif]

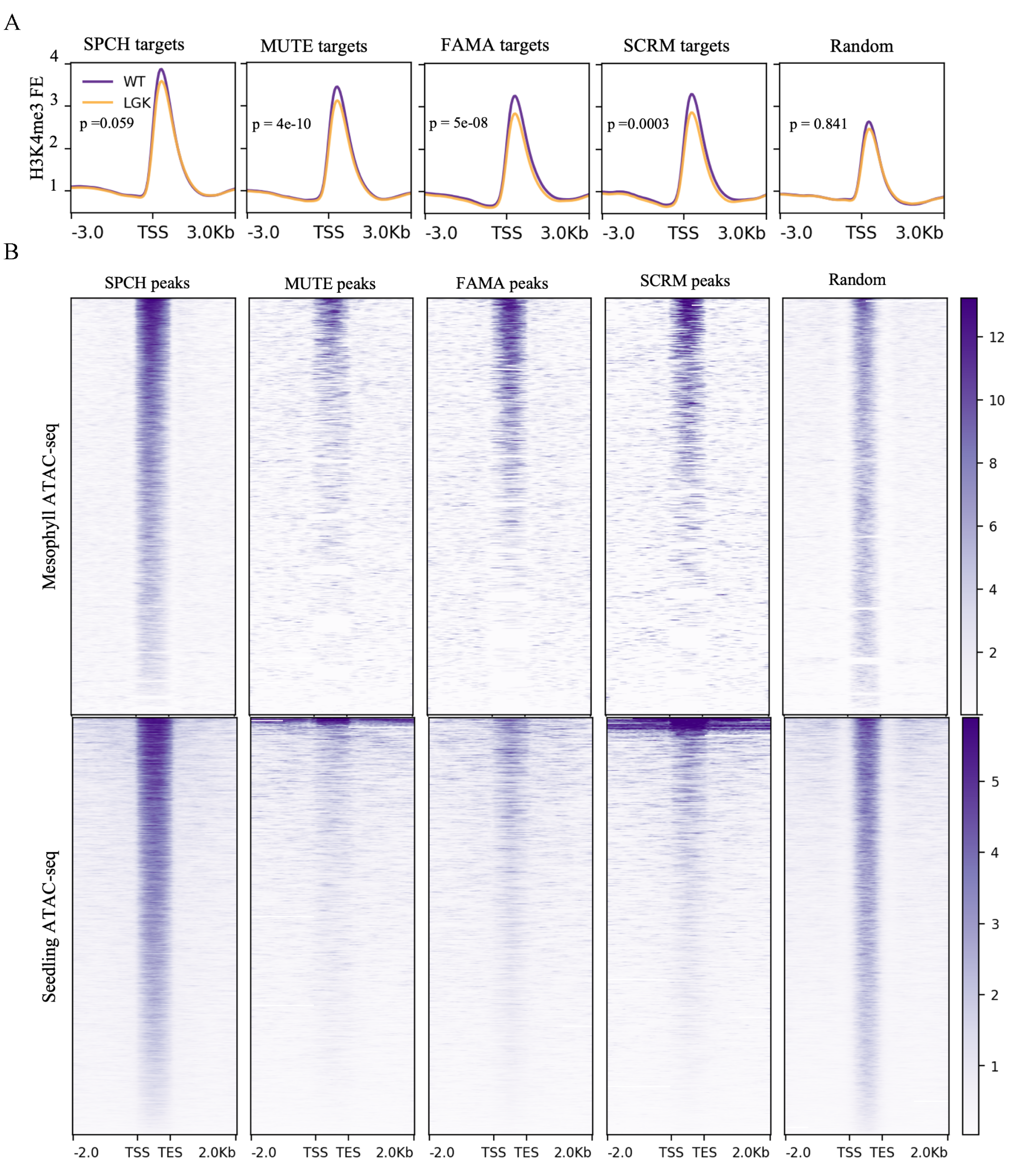

Supplement: S3 Fig — (A) FE of H3K4me3 levels in WT GC (purple) and FAMALGK GCs [37] (“pre-GCs” in orange) at targets of indicated stomatal bHLHs. (B) Chromatin accessibility of stomatal bHLHs’ binding sites and randomly sampled genomic intervals in mesophyll cells (top) and in whole seedlings (bottom) [34]. The data underlying this figure can be found in S1 Data. bHLH, basic helix–loop–helix; FE, fold-enrichment; GC, guard cell; SCRM, SCREAM; SPCH, SPEECHLESS; WT, wild type. (TIF) [file pbio.3002770.s003.tif]

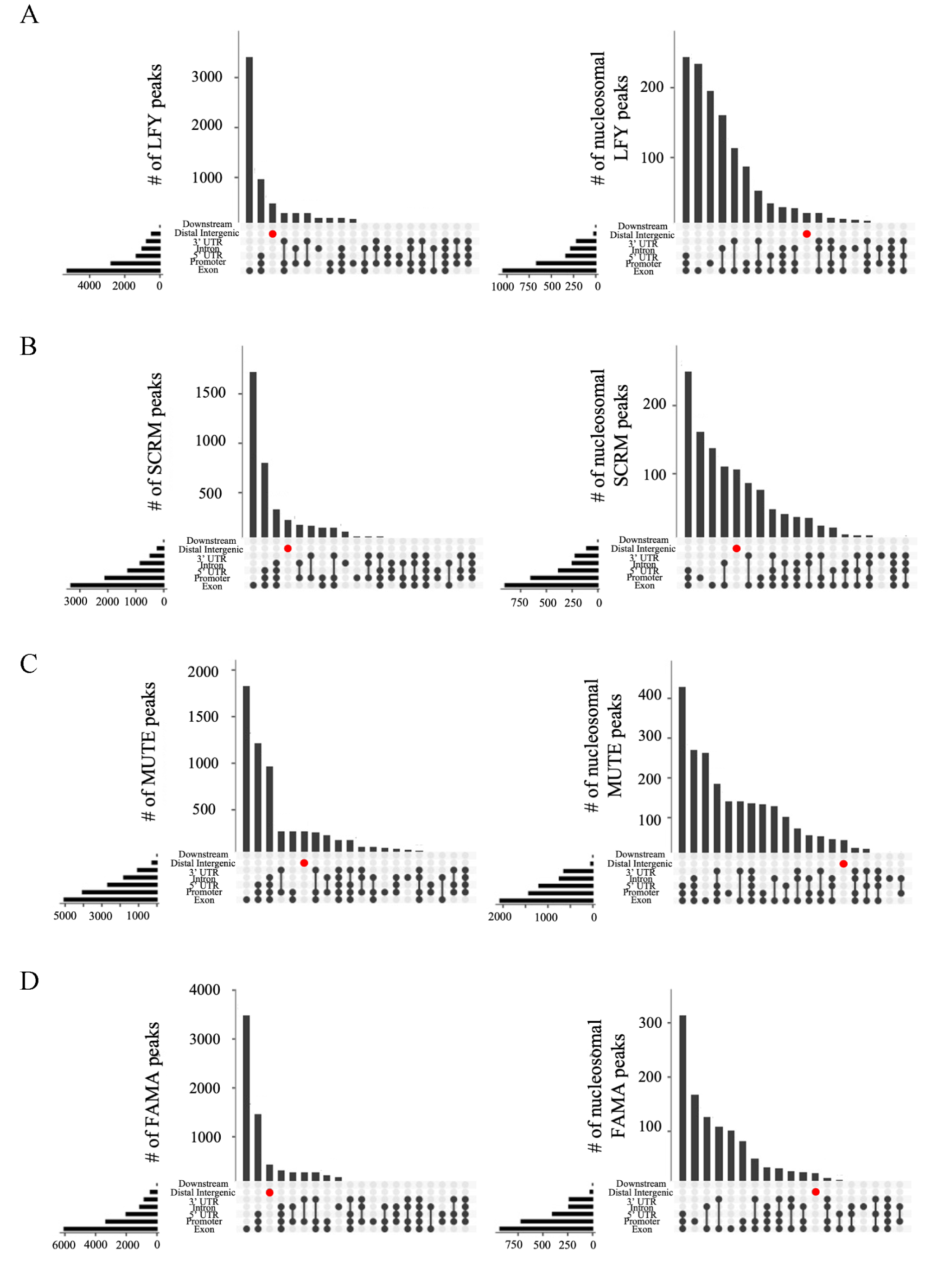

Supplement: S4 Fig — Histograms showing the distribution of all targets (left) and nucleosomal targets (right) of LFY (A) SCRM (B) MUTE (C) and FAMA (D). The red dot marks distal intergenic regions. The data underlying this figure can be found in S1 Data. LFY, LEAFY; SCRM, SCREAM; TF, transcription factor. (TIF) [file pbio.3002770.s004.tif]

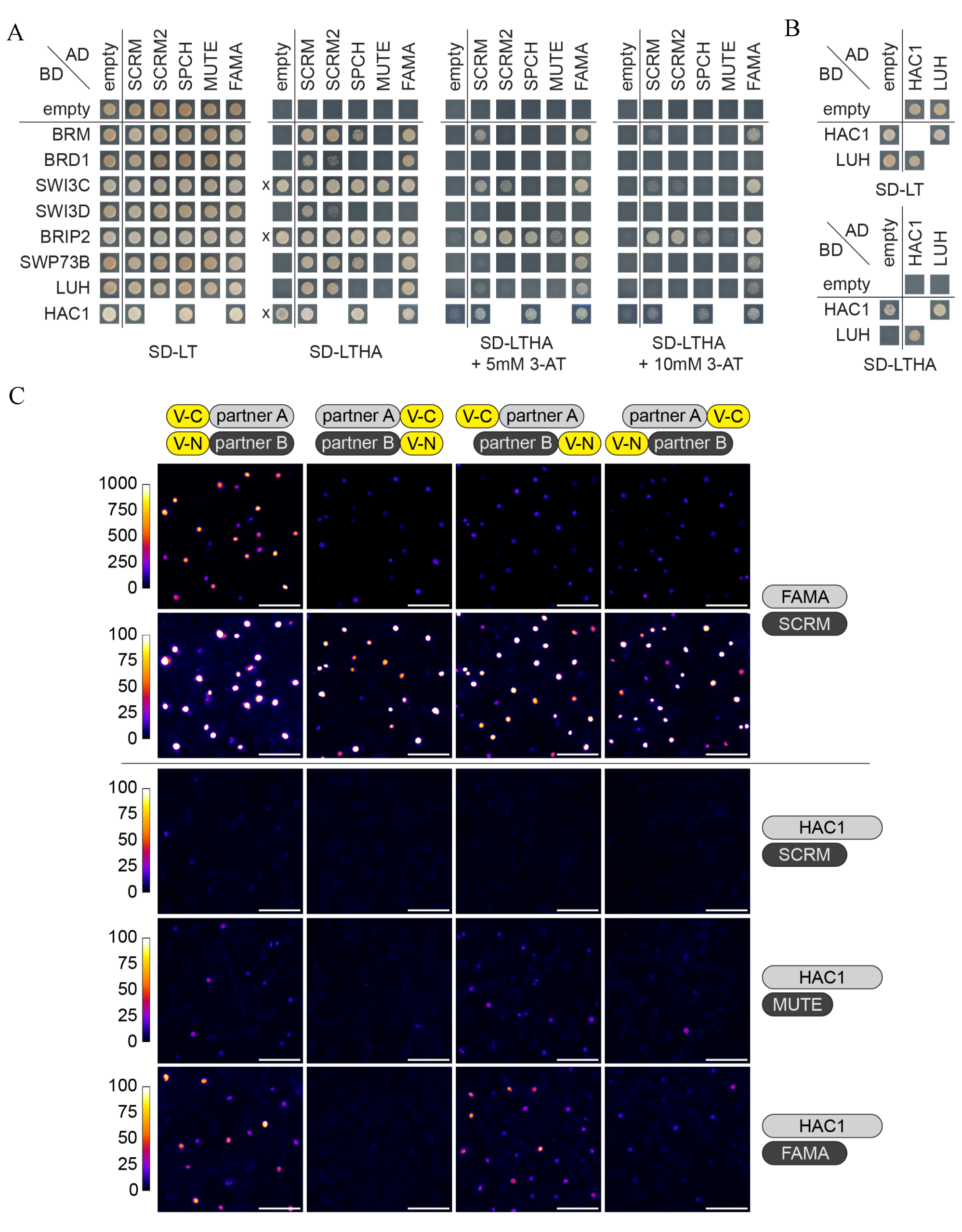

Supplement: S5 Fig — (A) Full panel of Y2H assays between AD-fused SCRM, SCRM2, SPCH, MUTE, and FAMA and BD-fused SWI/SNF components and HAC1. Yeast was spotted onto SD-LT to confirm cotransformation of the constructs and onto SD-LTHA to test for interaction. 3-AT was added to some SD-LTHA plates as indicated to overcome autoactivation of SWI3C, BRIP2, and HAC1 (indicated by x). Interactions with SCRM are also shown in Fig 3B. (B) Y2H of HAC1 and LUH. (C) FAMA and MUTE, but not SCRM, interact weakly with HAC1 via bimolecular fluorescence complementation (BiFC) in N. benthamiana. V-N: Venus N-terminal half, V-C: Venus C-terminal half. AD, activation domain; BD, binding domain; bHLH, basic helix–loop–helix; SCRM, SCREAM; SPCH, SPEECHLESS; SWI/SNF, SWITCH DEFECTIVE/SUCROSE NONFERMENTABLE; Y2H, yeast two-hybrid; 3-AT, 3-amino-1,2,4-triazole. (TIF) [file pbio.3002770.s005.tif]

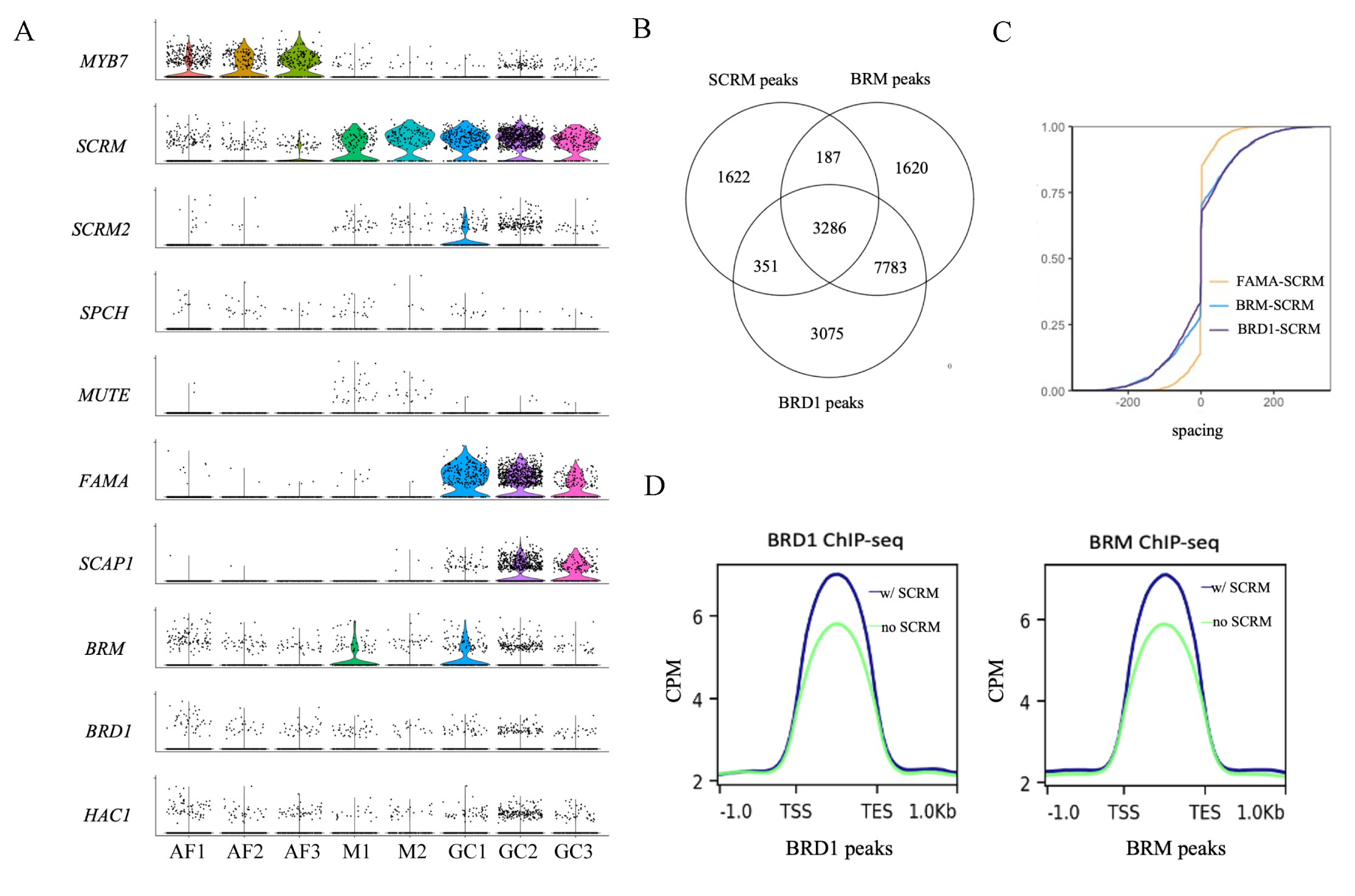

Supplement: S6 Fig — (A) Expression levels of BRM, BRD1, HAC1, and stomatal lineage markers in the scRNA-seq dataset from young developing true leaves. Cell type abbreviations are AF, alternative epidermal (likely pavement cell) fate; M, meristemoid; GC, guard cell. (B) A Venn diagram showing the overlap of ChIP-seq peaks of BRM, BRD1, and SCRM. (C) Cumulative plot of the distributions of spacing between indicated TF pairs shown in Fig 4D. (D) Average ChIP-seq signal (count per million reads) at BRD1 and BRM peaks that overlap with SCRM (blue) and those that do not (green). The data underlying this figure can be found in S1 Data. bHLH, basic helix–loop–helix; BRM, BRAHMA; ChIP-seq, chromatin immunoprecipitation followed by deep sequencing; SCRM, SCREAM; scRNA-seq, single-cell RNA-sequencing; TF, transcription factor. (TIF) [file pbio.3002770.s006.tif]

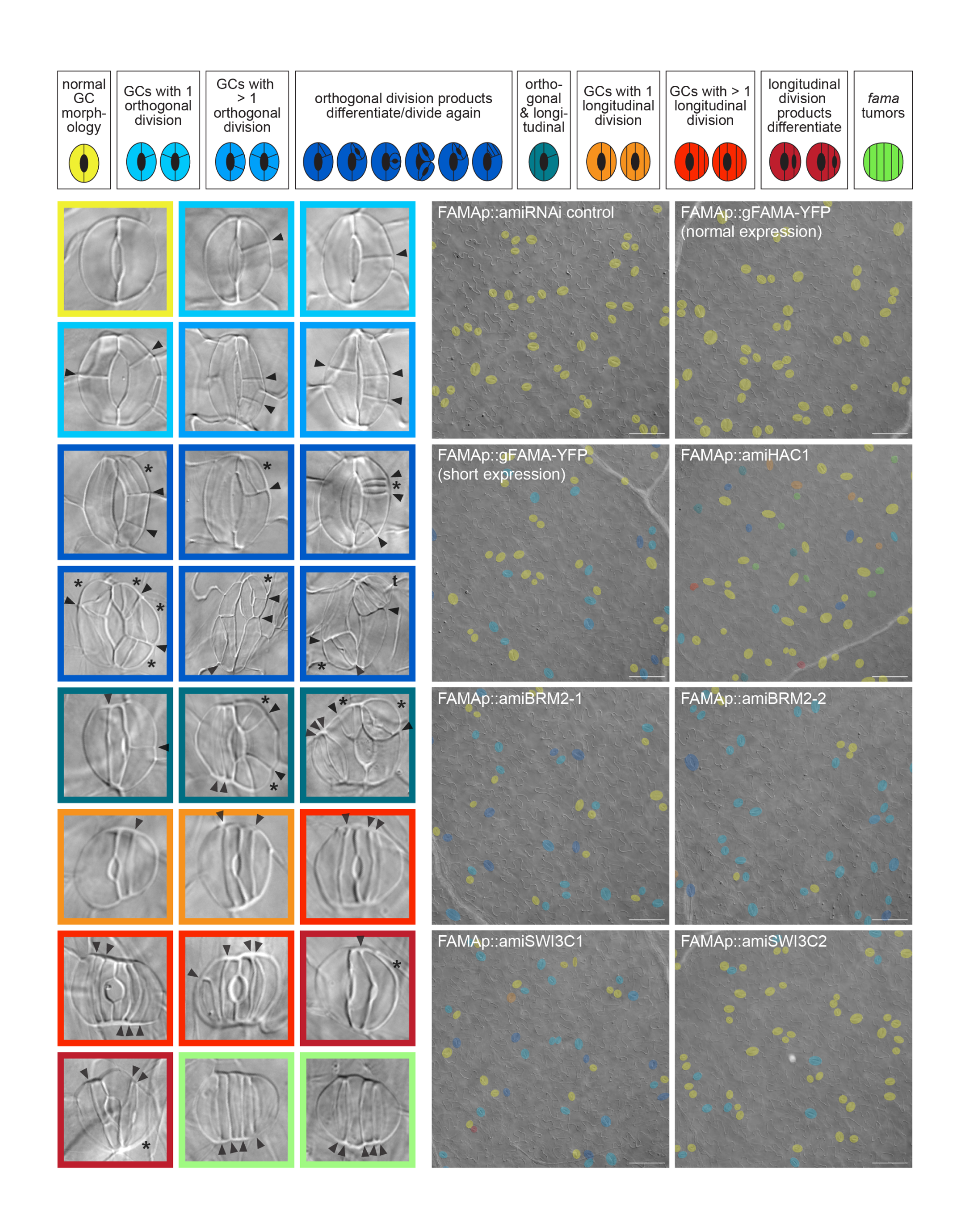

Supplement: S7 Fig — Top: GC variations observed in the lines described in Fig 5A are shown as simplified schemes in the top panel. Left: Examples for each class of GCs. Image borders are color-coded to match the top scheme. Transverse and longitudinal extra divisions in GCs are marked by filled and unfilled arrowheads, respectively. GCs within GCs are marked by an asterisk. Right: Zoomed out example images of the abaxial epidermis of the indicated lines showing the distribution of GCs with normal and abnormal morphology (overlays using the same color code as the top scheme). The images are DIC images of cleared 21-day-old cotyledons. Scale bar = 100 μm. (TIF) [file pbio.3002770.s007.tif]

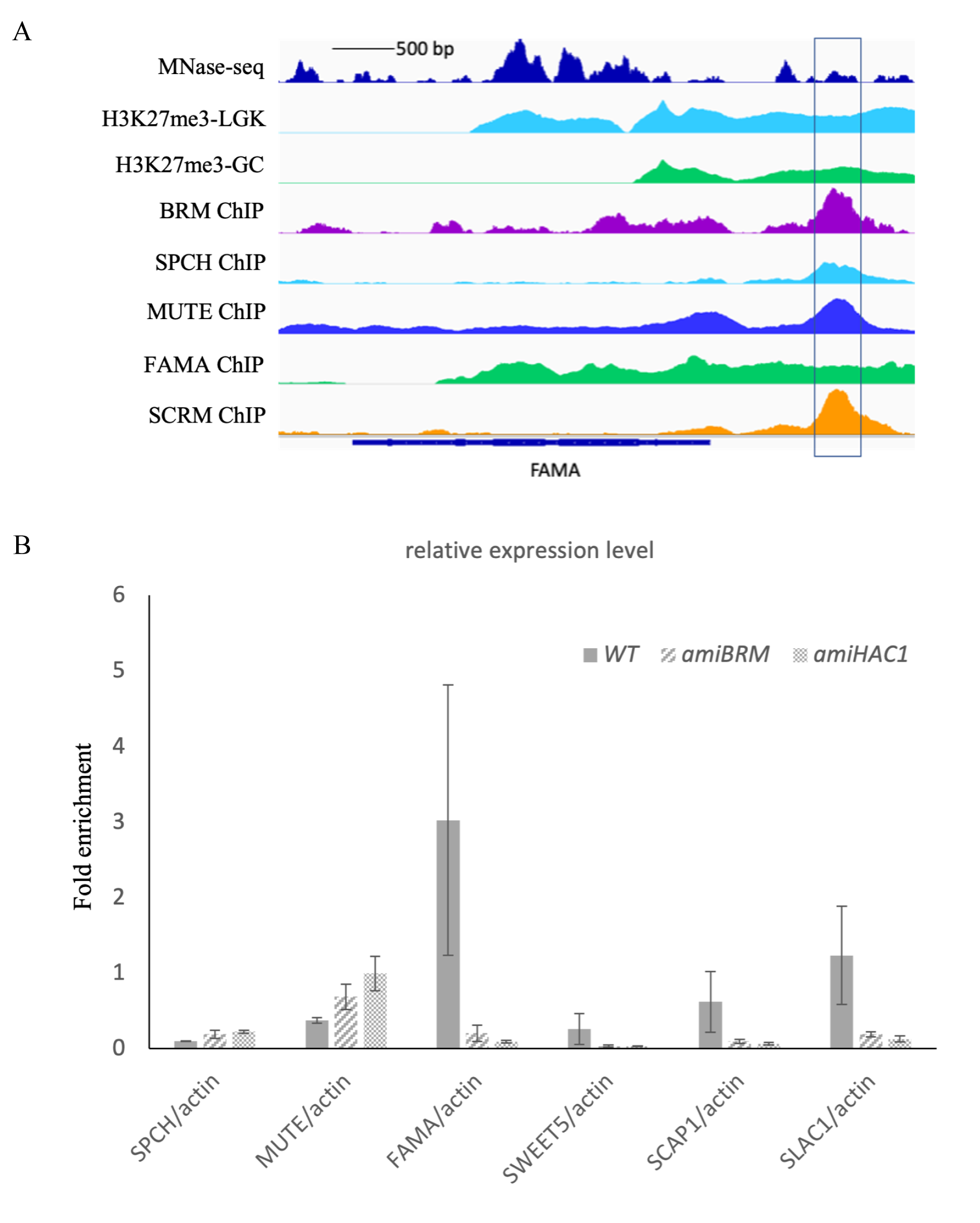

Supplement: S8 Fig — (A) View of the FAMA genomic locus, repressive H3K27me3 signal in “pre-GCs” (LGK, light blue) and GCs (green) and binding of BRM (purple) and the stomatal bHLHs (SPCH, light blue; MUTE, dark blue; FAMA, green; SCRM, orange) from ChIP-seq experiments. (B) Relative expression levels of SCRM-FAMA targets in FAMAp::amiBRM and FAMApro::amiHAC1 lines as assayed by qRT-PCR; RNA extracted from 14-day-old leaves and normalized to actin. The data underlying this figure can be found in S1 Data. bHLH, basic helix–loop–helix; BRM, BRAHMA; ChIP-seq, chromatin immunoprecipitation followed by deep sequencing; GC, guard cell; qRT-PCR, quantitative reverse transcription PCR; SCRM, SCREAM; SPCH, SPEECHLESS. (TIF) [file pbio.3002770.s008.tif]

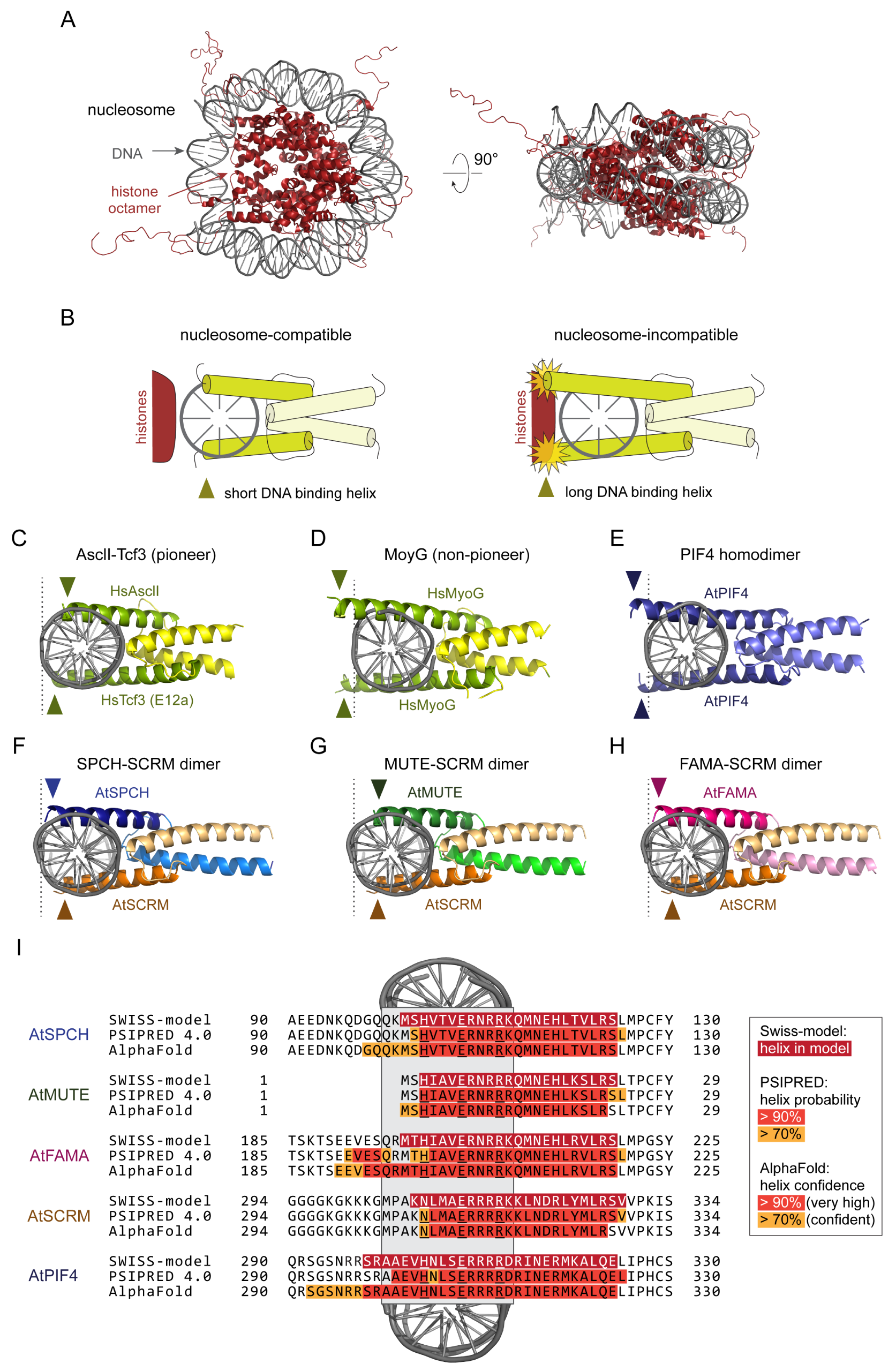

Supplement: S9 Fig — (A) X-ray crystal structure of the nucleosome core particle at 1.9 A resolution (PDB: 1kx5). The DNA and histone octamer are shown in grey and dark red, respectively. (B) Scheme of nucleosome-compatible and nucleosome-incompatible bHLH dimers. Histones do not interfere with binding of short DNA binding helices on the other face of the DNA (left), while binding of long DNA binding helices is obstructed (right). (C, D) Examples of human pioneer (Ascl1-Tcf3/E12a, C) and nonpioneer (MyoG, D) bHLH dimers, modelled with SWISS-model. The end of the DNA binding helix (dark green) is marked with an arrowhead. (E-H) 3D structures of plant bHLH heterodimers modelled with SWISS-model: PIF4 (E), SCRM-SPCH (F), SCRM-MUTE (G), SCRM-FAMA (H). The end of the DNA helices of the SCRM heterodimer is of similar length to the Ascl1-Tcf3 dimer and does not protrude beyond the DNA. The PIF4 heterodimer, in contrast resembles MyoG. (I) Comparison of the length of the DNA binding helix in the models shown in (E-H) with additional structure predictions obtained with PSIPRED 4.0 and AlphaFold2. Residues predicted to be part of the helix are highlighted in dark red (model), red (high confidence), and orange (medium confidence). The part of the helix that aligns with the DNA dimer is marked by a grey box. The 3 main residues that make contact with the DNA are underlined. Helix predictions for SCRM and MUTE are consistent between the 3 methods and always short. AlphaFold predicts an extension of the DNA-binding helix for SPCH and FAMA, which could interfere with nucleosome binding. Interestingly, in the PSIPRED prediction, this extension of the FAMA helix is interrupted by a stretch with very low helix probability, which could manifest as a flexible loop that breaks the rigid helix, thereby allowing nucleosome binding. bHLH, basic helix–loop–helix; SCRM, SCREAM; SPCH, SPEECHLESS. (TIF) [file pbio.3002770.s009.tif]
