# Supplementary material for: bHLH transcription factors cooperate with chromatin remodelers to regulate cell fate decisions during Arabidopsis stomatal development
Source: PLoS Biol. 2024 Aug 16;22(8):e3002770. doi: 10.1371/journal.pbio.3002770 (PMC11357106; doi:10.1371/journal.pbio.3002770)
Supplement: S2 Table — (DOCX) [file pbio.3002770.s011.docx]

**S2_Table.** **Primer list**

| **primer** | **experiment** | **sequence** |
| --- | --- | --- |
| SCRMp fw | PL | ACCACCGTCAATAACATCGTTAAGTAG |
| SCRMp rv | PL | CGCCAAAGTTGACACCTTTACCCC |
| SCRM no stop fw | PL, BiFC | CACCATGGGTCTTGACGGAAACAATGG |
| SCRM no stop rv | PL, BiFC | GATCATACCAGCATACCCTGCT |
| SCRM2 Y2H fw | Y2H | ACCAGAATTCATGAACAGCGACGGTGTTTGGC |
| SCRM2 Y2H rev | Y2H | CAAGGATCCTCAAACCAAACCAGCGTAACCTGCT |
| SPCH Y2H fw | Y2H | GTGAATTCATGCAGGAGATAATACC |
| SPCH Y2H rev | Y2H | GTGGATCCCTAGCAGAATGTTTGC |
| MUTE Y2H fw | Y2H | GTGAATTCATGTCTCACATCGCTG |
| MUTE Y2H rev | Y2H | TGGATCCTTAATTGGTAGAGACGATC |
| FAMA Y2H fw | Y2H | ACCAGAATTCATGGATAAAGATTACTCGGCACCAAACT |
| FAMA Y2H rev | Y2H | CAAGGATCCTCAAGTAAACACAATATTTCCCAGGTTAGAGC |
| BRM Y2H fw | Y2H | CACCATGCAATCTGGAGGCAGTGGC |
| BRM Y2H rev | Y2H | CAAGGATCCCTATAAATGGCTAGGCCGTCTTTTAC |
| BRD1 Y2H fw | Y2H | CACCATGGGTGAGGTAGCAGATACA |
| BRD1 Y2H rev | Y2H | CAGCTGTAATGCCAGATCCG |
| SWI3C Y2H fw | Y2H | CACCATGCCAGCTTCTGAAGATAGAAGAG |
| SWI3C Y2H rev | Y2H | CAAGGATCCTTAGTTTAAGCCTAAGCCGGACC |
| SWI3D Y2H fw | Y2H | CACCATGGAGGAAAAACGACGCGATTC |
| SWI3D Y2H rev | Y2H | CAAGGATCCCTAAACCGAAGAAACATTGTCTGAAC |
| BRIP2 Y2H fw | Y2H | CACCATGGAGGACACGAAGCCA |
| BRIP2 Y2H rev | Y2H | CCGGCTATTCAAATCAAATTCTCC |
| SWP73B Y2H fw | Y2H | CACCATGTCTGGTAACAACAACAATCC |
| SWP73 Y2H rev | Y2H | CCAACTCCCTGGCCCA |
| LUH Y2H fw | Y2H | CACCATGGCTCAGAGTAATTGGGAAG |
| LUH Y2H rev | Y2H | CTTCCAAATCTTTACGGATTTGTCATG |
| HAC1 fw | Y2H, BiFC | CACCATGAATGTTCAGGCTCACATGTCG |
| HAC1 stop rev | Y2H, BiFC | GAAGATCAGCCAACCAAACCCC |
| HAC1 no stop rev | BiFC | ACCTGAGCCCCCAGCGAC |
| SCRM stop BiFC rv | BiFC | TCAGATCATACCAGCATACCCTGCT |
| MUTE BiFC fw | BiFC | CACCATGTCTCACATCGCTGTTGAAAGGAATCG |
| MUTE no stop BiFC rv | BiFC | ATTGGTAGAGACGATCACTTCATCAGAC |
| MUTE stop BiFC rv | BiFC | TTAATTGGTAGAGACGATCACTTCATCA |
| FAMA BiFC fw | BiFC | CACCATGGATAAAGATTACTCGGCACCAAA |
| FAMA no stop BiFC rv | BiFC | TCAAGTAAACACAATATTTCCCAGGTTAG |
| FAMA stop BiFC rv | BiFC | AGTAAACACAATATTTCCCAGGTTAGAGC |
| amiHAC1 1-I | amiRNA | gaTACATTTATCACATTGGACCCtctctcttttgtattcc |
| amiHAC1 1-II | amiRNA | gaGGGTCCAATGTGATAAATGTAtcaaagagaatcaatga |
| amiHAC1 1-III | amiRNA | gaGGATCCAATGTGAAAAATGTTtcacaggtcgtgatatg |
| amiHAC1 1-IV | amiRNA | gaAACATTTTTCACATTGGATCCtctacatatatattcct |
| amiHAC1 2-I | amiRNA | gaTTTATCACATTGGACCCACGAtctctcttttgtattcc |
| amiHAC1 2-II | amiRNA | gaTCGTGGGTCCAATGTGATAAAtcaaagagaatcaatga |
| amiHAC1 2-III | amiRNA | gaTCATGGGTCCAATCTGATAATtcacaggtcgtgatatg |
| amiHAC1 2-IV | amiRNA | gaATTATCAGATTGGACCCATGAtctacatatatattcct |
| amiHAC1 3-I | amiRNA | gaTAGCAATTTTAAACAGGCCCTtctctcttttgtattcc |
| amiHAC1 3-II | amiRNA | gaAGGGCCTGTTTAAAATTGCTAtcaaagagaatcaatga |
| amiHAC1 3-III | amiRNA | gaAGAGCCTGTTTAATATTGCTTtcacaggtcgtgatatg |
| amiHAC1 3-IV | amiRNA | gaAAGCAATATTAAACAGGCTCTtctacatatatattcct |
| amiHAC1 4-I | amiRNA | gaTACCTATAATTGAGCCTGCAGtctctcttttgtattcc |
| amiHAC1 4-II | amiRNA | gaCTGCAGGCTCAATTATAGGTAtcaaagagaatcaatga |
| amiHAC1 4-III | amiRNA | gaCTACAGGCTCAATAATAGGTTtcacaggtcgtgatatg |
| amiHAC1 4-IV | amiRNA | gaAACCTATTATTGAGCCTGTAGtctacatatatattcct |
| amiBRM 1-I | amiRNA | gaTACAAATTTGCGGTACGCCCTtctctcttttgtattcc |
| amiBRM 1-II | amiRNA | gaAGGGCGTACCGCAAATTTGTAtcaaagagaatcaatga |
| amiBRM 1-III | amiRNA | gaAGAGCGTACCGCATATTTGTTtcacaggtcgtgatatg |
| amiBRM 1-IV | amiRNA | gaAACAAATATGCGGTACGCTCTtctacatatatattcct |
| amiBRM 2-I | amiRNA | gaTACTCTAGTCTTTCTACGCGGtctctcttttgtattcc |
| amiBRM 2-II | amiRNA | gaCCGCGTAGAAAGACTAGAGTAtcaaagagaatcaatga |
| amiBRM 2-III | amiRNA | gaCCACGTAGAAAGAGTAGAGTTtcacaggtcgtgatatg |
| amiBRM 2-IV | amiRNA | gaAACTCTACTCTTTCTACGTGGtctacatatatattcct |
| amiSWI3C 1-I | amiRNA | gaTTAACGCTTGCAATTGCGCAAtctctcttttgtattcc |
| amiSWI3C 1-II | amiRNA | gaTTGCGCAATTGCAAGCGTTAAtcaaagagaatcaatga |
| amiSWI3C 1-III | amiRNA | gaTTACGCAATTGCATGCGTTATtcacaggtcgtgatatg |
| amiSWI3C 1-IV | amiRNA | gaATAACGCATGCAATTGCGTAAtctacatatatattcct |
| amiSWI3C 2-I | amiRNA | gaTGATTTCATCCTATCGTCCGCtctctcttttgtattcc |
| amiSWI3C 2-II | amiRNA | gaGCGGACGATAGGATGAAATCAtcaaagagaatcaatga |
| amiSWI3C 2-III | amiRNA | gaGCAGACGATAGGAAGAAATCTtcacaggtcgtgatatg |
| amiSWI3C 2-IV | amiRNA | gaAGATTTCTTCCTATCGTCTGCtctacatatatattcct |
| amiSCRAMBLE F | amiRNA | CACC CCCAAACACACGCTCGGACGC |
| amiSCRAMBLE R | amiRNA | GCCGCTCTAGAACTAGTGGATCC |
| FAMA qPCR F1 | qPCR | ACAACAGCAGCAACATCAACT |
| FAMA qPCR R1 | qPCR | GCATGAGATGTTGGGTTTGGT |
| FAMA qPCR F2 | qPCR | CCAACGGATGACTCATATCGC |
| FAMA qPCR R2 | qPCR | TCATGTCCCTACCGGTTTCTC |
| SCAP1 qPCR F1 | qPCR | CAAACCAAGAAACCACCGTCT |
| SCAP1 qPCR R1 | qPCR | ATGGGGACGTTTCTTAGAGCA |
| SCAP1 qPCR F2 | qPCR | GAAACTGTTGTCGTCGAGAGG |
| SCAP1 qPCR R2 | qPCR | TCACTTCCTCCTCCTCCTGTA |
| SLAC1 qPCR F1 | qPCR | ACGCTCAGCAAACAAAAGTCT |
| SLAC1 qPCR R1 | qPCR | CCTTAGGAGAAACGGCCATTG |
| SLAC1 qPCR F2 | qPCR | GACCAAACCGAGGGAAACAAA |
| SLAC1 qPCR R2 | qPCR | CTCCACCGTTGATGATTCCAC |
| SLAC1 qPCR F3 | qPCR | GTTGTAGGGAATTTCGTCGGG |
| SLAC1 qPCR R3 | qPCR | GGGGCAGCAATGAACATAGAG |
| ACT2 qPCR F1 | qPCR | TGTCTCGTTGTCCTCCTCACT |
| ACT2 qPCR R1 | qPCR | ACAAGATCGAGATCCAGCAAA |
| ACT2 qPCR F2 | qPCR | GTTGGGATGAACCAGAAGGAT |
| ACT2 qPCR R2 | qPCR | GAGGAGCCTCGGTAAGAAGAA |
| MUTE qPCR F1 | qPCR | CACATCGCTGTTGAAAGGAAT |
| MUTE qPCR R1 | qPCR | GTCGGTTTAGGGTCTTTCGAC |
| MUTE qPCR F2 | qPCR | GTTGTCTCTAGGCGAATCGTG |
| MUTE qPCR R2 | qPCR | TCAAGAGTTAGCTCCTCCAAGC |
| SPCH qPCR F1 | qPCR | TGAAGGTGCCGGAGAGATATC |
| SPCH qPCR R1 | qPCR | CCGTCTCCGTCTTCTTCTTCT |
| SPCH qPCR F2 | qPCR | TCCCGGGACAAGTTATGAAGA |
| SPCH qPCR R2 | qPCR | TGCTGAATTTGTTGAGCCAGT |
| SWEET5 qPCR F1 | qPCR | TCCGAGTTTAAGCCAGATCCA |
| SWEET5 qPCR R1 | qPCR | GCGAAGACAAAGAAGATGGTGA |
| SWEET5 qPCR F2 | qPCR | CGTTTGGGTCATTTATGCATGTC |
| SWEET5 qPCR R2 | qPCR | CTATCAAGCCTGGCCAAGTTC |
