# Supplementary material for: bHLH transcription factors cooperate with chromatin remodelers to regulate cell fate decisions during Arabidopsis stomatal development
Source: PLoS Biol. 2024 Aug 16;22(8):e3002770. doi: 10.1371/journal.pbio.3002770 (PMC11357106; doi:10.1371/journal.pbio.3002770)
Supplement: Attachment — Submitted filename: Liu_Mair_Plos_RRv1_response_final.docx [file pbio.3002770.s013.docx]

Response to reviewers

Original comments in black text, our responses in blue.

Editor’s summary and comments

The reviews are attached below. As you will see, the reviewers find the conclusions novel and interesting for the field, but they also raise several issues that would need to be addressed before we consider the manuscript for publication. Reviewer 1 mostly suggests minor improvements in the figurers and asks for several clarifications, whereas Reviewer 2 is more critical and suggests an experiment expressing FAMA to see if it has a similar effect to MUTE. While this reviewer could have misinterpreted the arguments considered, we think this experiment would be important to clarify whether or not the differences in binding profile between MUTE and FAMA translate into differences in the ability to displace nucleosomes. In addition, the Academic Editor thinks that the section describing the differences between FAMA and MUTE is hard to interpret, thus we would encourage you to improve the writing to make the argument clearer to readers. The Academic Editor is also concerned that the differences between FAMA and both MUTE and SCRM might be technical because SCRM and MUTE profiles come from previous datasets, whilst FAMA profile is from a database generated for this manuscript. Thus, we think you should present in the main results section some data to confirm that the new profiling data is comparable – in terms of coverage, signal to noise, background signal, number of peaks, etc. In an ideal situation, the MUTE or SCRM profiling could be repeated to demonstrate that technical variability does not account for the differences between their profiles and that of FAMA.

We thank the editor and reviewers for critical comments and clear guidance on what needs to be revised.

From the editors, we see two main issues to address (1) clarification of MUTE vs. FAMA behaviors and (2) deeper consideration of whether the diverse ChIP-seq experiments compared in this work could lead to artifacts. Because point (1) is also raised by reviewers, we will address that in our response to reviewers. Below we provide more extensive rationale and data to address the ChIP-seq experiments.

**Response:**

**Standards in the field for TF ChIP experiments**

In plants, TF-ChIP seq is still a bespoke practice as there are few antibodies available for proteins and so most experiments are performed by making transgenic epitope tagged TFs. There is a history of others in the field successfully reanalyzing datasets from multiple sources (eg: Wang et al., Genome Res. 2015. 25: 246-256. Xiao et al., Nat Genet. 2017. 49, 1546–1552).

In the case of developmental regulators, ideally these experiments are performed with the native promoters and with the transgene rescuing the mutant version of the TF gene of interest. This makes the experiments low-throughput. In the case of SPCH, MUTE and FAMA, these proteins are all expressed in the epidermis of young leaves but are in distinct cell types. Thus, while we understand the desire to match tissues at closely as possible, and we do this, ultimately, these TFs do not normally bind their targets in exactly the same cells. The fact that SPCH, MUTE and FAMA act sequentially in cells with potentially different chromatin states is one of the most interesting parts of the biology of the system.

Nonetheless, we agree that we should be wary of differences that could arise because of technical variability. The datasets used in this paper are created in different labs and have different coverages and signal- to-noise ratios. However, all meet the thresholds for saturation of the (small) Arabidopsis genome, thus there is minimal concern that the differences in the binding profiles are caused by genome coverage.

**Could differential peaks result from technical differences in ChIPs?**

We considered the question of whether “differential peaks” for the TFs could result from purely technical reasons rather than biological ones. For this we needed to think about what artifacts a “bad ChIP” produces. Reading across the literature and looking at our own experiments over the years, we noticed that the hallmarks of low-quality ChIP was that they could only identify the most easily ChIP’d regions – that is they result in false negatives in specific binding sites rather than false positives in ectopic sites.

To assess technical artifacts in the data used specifically in this paper, we looked at the ChIP-seq peaks shared across the datasets. We find some “hyperChIPable” regions shared in all of the datasets, including the non-transgenic controls, and these likely represent technical issues such as regions of local chromatin easily sonicated or “sticky” in the ChIP process. These regions (labeled “hyper” in figure R1a & b, will be referred as hyperChIPable or HC peaks). We then used the behavior of these peaks to define a metric with which to evaluate the behavior of individual datasets.

In the MUTE ChIP dataset (Figure R1a), which has the lowest signal to noise ratio, the HC peaks (hyper) are notably higher than the “real” MUTE peaks (all) whereas in FAMA (figure R1b), which was the highest quality ChIP the real peaks have higher signal enrichment, which is close to, yet still lower than, the HC regions.

**Figure R1: ChIP-seq fold enrichment of MUTE (a) and FAMA (b) at all peaks (green) and hyper-ChIPable regions (blue). c) Examination of data from SCRM ChIP-seq. The ChIP-seq fold enrichment of SCRM at peaks that are uniquely shared between SCRM and each of SPCH, FAMA or MUTE are shown in dark blue, light blue and yellow, respectively.**

We then took advantage of the fact that SPCH, MUTE and FAMA are each partners with SCRM. Thus, there should be biologically meaningful peaks shared between SCRM and each of SPCH, MUTE and FAMA. These peaks are discovered by comparing SCRM occupancy (from the SCRM ChIP experiment) to SPCH, MUTE and FAMA occupancy in their respective ChIPs. Some of these peaks are unique to each of these pairs (ie. some MUTE-SCRM shared peaks are different from FAMA-SCRM shared peaks).

We can then assess the behaviors of each set of shared peaks in a common experiment--the SCRM ChIP dataset. If a peak arose purely from technical issues (e.g., lower affinity chromatin could be captured in the FAMA ChIP but not in the MUTE ChIP) then in the SCRM-ChIP the “FAMA-SCRM” regions in aggregate would have lower ChIP signal enrichment than “MUTE-SCRM” regions. This is not the case. In Figure R1c, we can see that the in the SCRM ChIP, SPCH-SCRM, MUTE-SCRM and FAMA-SCRM peaks all have similar signal enrichment. The enrichment in each case is different (lower) that for the hyper peaks (similar to what is already plotted in R1a and R1b). Based on these types of comparisons we conclude that the vast majority of the TF-specific peaks we identified here are not due to technical variation in the ChIP process but result from biologically meaningful differences in TF occupancy in chromatin.

**Specific responses to reviewers' comments**

Rev. 1:
The manuscript by Liu et al, "Cell Fate Programming by Transcription Factors and Epigenetic Machinery in Stomatal Development", investigated how MUTE, SCRM and PAMA, key bHLH TFs in stomatal development, interplay with chromatin at their target genes and how they functionally interplay with other epigenetic factors to control gene expression.

The authors first analyzed transcriptional entropy in stomatal lineage single cell RNA-seq data, and identified different phases of transcriptional regulation during stomatal development, which they intended to use as indicators of differentiated vs undifferentiated cells . They further showed that MUTE, FAMA and SCRM could indeed overcome repressive chromatin and each has the potential to induce nucleosome repositioning during these phases. Lastly, the team used TurboID-based strategy to identify the proteins that are physically associated with FAMA by LC-MS and found BRM complex components and HAC1, a histone acetyl transferase. They then demonstrated that stomatal lineage-specific knock down of components of the BRM complex or HAC1 results in reduced expression of the bHLHs' targets and plants that are impaired in forming terminally differentiated stomata. It is a work of significant interest to the plant biology and epigenetics community; and in my view, the major contribution of this work is the identification of BRM and HAC1 and demonstration of their physical association with the bHLHs and their functional interplay at target genes in controlling stomata development. Overall, the manuscript is well written and the evidence presented support their conclusion.

I only have a few minor suggestions for improving the manuscript:

Figure 1a: what is the X-axis? Please label or explain.

**Response:**
We apologize that the X-axis description was cut off in the first version of the figure. In the now complete figure, the X-axis is the number of genes expressed in hundreds, i.e. x =5 means 500 genes expressed.

Lines 147-156 and Figure 2 and Figure S3: the authors analyzed and compared previously published
ChIP-seq data with their newly generated ChIP-seq data, as well as scRNA-seq data. Please add some background information indicating that these experiments were conducted with similar plant materials including growth conditions so they are comparable and they are suitable for this manuscript. This also applies to other places in this manuscript where published data were used.

**Response:**
All ChIP data are generated from in seedlings grown at standard growth condition for Arabidopsis except for LFY, which was done in root explants. Single cell transcriptional profiles were generated from leaf seedlings from which the epidermis was FACS isolated or from seedling roots (for entropy proof of concept). **This information is now included in lines 452-454**.

Table 1: about the TurboID-based MS identification of SCRM interactors: should the numbers of peptides identified by MS for each of the interactors listed? Such data would not only show the interaction, but can also show it in a quantitative manner.

**Response**:

Thank you for this suggestion. We have included an excel file as **Supplementary Table 1** that contains information about the MS analysis results for readers with a more in-depth interest.

Peptide or spectral counts are often shown to support interaction strength in MS data. We originally opted not to include such information because of the cell-type specific nature and generally low abundance of SCRM. While with a highly/ubiquitously expressed proteins one could expect to detect hundreds of spectral counts, with a rare bait such as SCRM or other rare TFs, we often get 20 or less peptide/spectral counts for the bait itself and even fewer for most interacting candidates. The number of identified peptides is therefore less meaningful for estimating relative interaction strength with different putative partners.

For our enrichment analysis, we did not rely on peptide counts, as they are not very quantitative and also highly biased for protein size. We implied MaxQuant’s label free quantification (LFQ) algorithm, which integrates MS1 level information about peptides selected for MS2 fragmentation (identification), and such that were not selected for fragmentation but have the same characteristics (e.g. retention time), to overcome limitations of MS resolution and to calculate protein abundance in all samples more accurately.

In our supplementary table we therefore provide both spectral count information for each sample (plus information in molecular weight, total number of identified peptides for each protein and sequence coverage) and the results of our enrichment analysis using LFQ-based quantification (log2-fold changes, p-values and q-values for all comparisons between SCRM-TurboID and the control groups).

Lines 241-244: the authors said "Interaction between the bHLHs and HAC1 was relatively weak in both Y2H (Fig S5a) and bimolecular fluorescence complementation (BiFC) assays (Fig S5c), suggesting that strong interaction with HAC1 may require the presence of the heterodimer or an additional linker protein (e.g. LUH, which interacts both with SCRM and FAMA and with HAC1 (Fig S5a and b))." This statement is true for Y2H, but not for BiFC as it is done in planta.

**Response**:
We agree that it is more likely for BiFC to work without adding all the required binding partners. An ortholog of LUH is likely present in *N. benthamiana* leaves and could act as a bridge between the bHLHs and HAC1.

However, we feel that it is still true that we cannot assume that all co-factors would be present just because BiFC is done in plants because these cells are not the same identity as the Arabidopsis early stomatal lineage cells in which the interactions would take place. If the interaction requires a functional dimer (potentially with an additional co-factor or in the context of DNA binding), just co-expressing HAC1 and one of the bHLH partners may not be sufficient. SPCH, MUTE, FAMA and SCRM are only expressed at specific stages of stomatal development in Arabidopsis (and SCRM also under cold stress conditions). The same is likely the case in *N. benthamiana*. We can therefore not assume that the SCRM or FAMA ortholog of *N. benthamiana* is expressed at the time of imaging nor do we know if this would be the preferred dimerization partner in this system and under these conditions.

Figure 5 and the related text: about the BRM and HAC1 amiRNAi lines, the authors did not show in this manuscript to what extent the BRM/HAC1 transcript or protein were reduced? It would be helpful to present such data. It seems that they were using their previously generated lines, but it would be helpful to confirm again, as it might change over time. However, I did notice that in the Materials and Methods section, they described the RNA extraction and qRT-PCR analysis of these lines, but I did not see any mentioning of such results in either the figures or the main text.

**Response:**

We generated HAC1 amiRNA lines specifically for this manuscript, and now provide additional information about their behavior. We agree that it’s important to assess whether an amiRNA does in fact reduce its target. This becomes somewhat challenging when the target is expressed in many cells and the amiRNA is targeted to rare cell types, as we do in this paper. Nonetheless, using qRT-PCR analysis we can show a reduction in HAC1 (Figure R2). Because the TMM promoter expresses the HAC1 amiRNA in only 1-5% of the leaf cells used in this experiment, the consistent reduction of *HAC1* RNA suggests a quite strong reduction in the target cells.

Figure R2: RT-qPCR of normalized expression levels of the *HAC1* gene in control (ML1, which is the ML1p::RCI2A-YFP line we used for transforming the amiRNAs) and 4 different amiRNAs that target *HAC1*. 3 pairs of primers (F1+R1, F2+R2, F3+R3) that target 5’, middle and 3’ of the gene body were used. The amiRNAs are driven under TMM promoter to target all early stomatal lineage cells.

Line 298: cite references for "…a line we termed 'short-lived FAMA…',".

**Response:**

This line was generated for this manuscript and is described in the text in **lines 295-297** and in the materials and methods lines 436-438: “The short-lived FAMA line is an individual line that harbors the same construct as FAMApro::gFAMA-YFP/fama-1 [62] but expresses gFAMA-YFP for a shorter period of time in each developing GC.”

Rev. 2:
The ms by Liu et al. on cell fate reprogramming by TFs and their recruitment of epigenetic machinery in stomata provides thoughtful experiments on a key aspect of plant gene regulation—the identification of pioneering transcription factors that can access closed chromatin. This is a key mechanism in development that determines how cells embark on new fates. The paper contains a series of key experiments that provide evidence for what may be perhaps the second good case for pioneering transcription factors in plants. The experiments themselves are rigorous and appropriate tests for this major claim. The argument is strong, and, overall, the chain of evidence, the logic of the experiments, and the presentation and interpretation of the results are all excellent. I have one major question on the chain of evidence that I cannot reconcile and weakens the argument if my interpretation is correct. It may not be, in which case, I feel the ms needs some clarification. I also have several minor and easily addressed comments.

1. In the section on bHLHs having the potential to bind closed chromatin and remodel it (starting 178), the evidence showed that FAMA (but not SCRM or MUTE) had comparable affinity to nucleosome and non-nucleosome bound chromatin. This, together with binding co-occurrence data led to the conclusion that FAMA and SCRM together bind nucleosomal DNA. But then, in experiments that built on the evidence to show the potential to remodel, the authors focused on MUTE. The inducible MUTE experiment followed by MNase nucleosome occupancy analysis supported its role to change nucleosome density. To me, this was a disconnect. The binding affinity pointed to FAMA as the pioneer factor with SCRM implicated as a partner. But then the functional test was done on MUTE, for which affinity evidence did not implicate? So, this breaks the chain of evidence in my mind that build the case for FAMA as a pioneering factor. The remodeling test would need to be done on FAMA, not MUTE? Could the authors address this issue?

**Response:**
Our data suggest SCRM, MUTE and FAMA may all have nucleosomal DNA binding potential. MUTE and SCRM behavior most closely resembles the canonical pioneer behavior of displaying higher affinity for DNA that is not wrapped around nucleosomes compared to nucleosomal regions and both SCRM and MUTE show that trend. FAMA comparable affinity at those two types of binding sites, which is actually non-canonical (though not unheard of in animal systems). We agree that in the ideal scenario, we would have nucleosome profiles before and after induction of each of the stomatal bHLH TFs.

We attempted these experiments, generating lines to induce expression and to capture a phenotypic effect, unfortunately in our hands, such experiments only robustly and reproducibly induced MUTE expression and measurable phenotypic consequences at the stage we used for all the ChIP experiments. Thus, we only pursued the nucleosome remodeling question with the MUTE induction. We think that this is a novel and important demonstration that a stomatal bHLH can bind nucleosomal regions and that the binding can have a functional consequence. We do wish we were able to also show this for FAMA, but repeated attempts to induce high, yet temporally defined expression, with available genetic tools did not produce behaviors that we thought would give us the needed resolution for the experiment. **We now mention this explicitly in the text (lines 215-218). We also moved some of the text describing FAMA behavior to the discussion.**

Minor Points
2. I struggled to try to understand how the entropy was measured until I carefully read the methods. But it's such a lengthy part of the set up that I think it should be clear in the text. E.g., I wondered whether entropy was being measured from perspective of a single gene among all the cells or among all the genes. The seems to be the case but clear explanation in the main text would help.

**Response:**
The entropy measurement is based on all the genes. **We have clarified this in the main text (lines 118-121).**

3. Fig. 2D, it looks like SPCH should be significant in the opposite direction from MUTE, FAMA, and SCRM. I couldn't see how these statistics were done, one tailed?

**Response:**

The statistics were done based on DE-seq package. We treated H3K27me3 signal pile up similar to RNA-seq data. H3K27me3 signals that fall into a gene body are treated as “expression counts” and the corrected p value was calculated using the DE-seq package and represented in the figure.

We think the reviewer may be noting the dip in SPCH 5’ of the TSS, and this keen observation made us realize we need to be clearer about what is considered in the analysis—differential enrichment is only calculated using the signal from the gene body (between TSS and TES). **We have now noted this explicitly in the legend of Figure 2.** As for the explanation for that “dip”, we did notice that SPCH targets in general are depleted in H3K27me3 compared to H3K27me3 levels at the targets of other bHLHs which contributes to an overall different background level. This could be interesting for future experiments, but is not germane to the analysis here.

4. I got a bit hung up on the FAMA_LGK experiment. It's not clear what is progression of events is assumed to be happening in the reprogramming. Are cells never reaching the mature state or do they get there and slip back, such that remodeling would have occurred and then reverted?

**Response:**
This is a good question. When we originally reported the FAMA_LGK “reprogramming” phenotypes (Matos et al., 2014) the experimental techniques available at that time led us to conclude that the cells had become guard cells, but then later reverted. The newer analysis in this paper, where we also include some additional measure of maturity and function, suggests that we may need to revise our assessment. Based on failure to (1) turn off ML1 and (2) failure to produce autofluorescent compounds typical in mature cell pores, it is more likely that the guard cells never completely reached the mature state.

Inspired by this reviewer’s question, we re-examined the older FAMA-LGK lines and found their GCs also did not turn off ML1 nor did they produce autofluorescent compounds, so it’s likely that they too never become fully differentiated. It becomes a bit of a philosophical point of what it means then to “revert” vs. never fully differentiate. We didn’t see a natural place to include this information in the current manuscript, so we are reporting this just for the reviewer’s interest, and also with the understanding that the journal’s review process, information in this response will be available to other readers.
